# Trachoma in Western Equatoria State, Southern Sudan: Implications for National Control

**DOI:** 10.1371/journal.pntd.0000492

**Published:** 2009-07-28

**Authors:** Lucia W. Kur, Diana Picon, Obec Adibo, Emily Robinson, Anthony Sabasio, Tansy Edwards, Aggrey Ndyaba, John Rumunu, Karinya Lewis, Mounir Lado, Jan Kolaczinski

**Affiliations:** 1 Ministry of Health, Government of Southern Sudan, Juba, Southern Sudan; 2 Malaria Consortium – Southern Sudan Office, Juba, Southern Sudan; 3 Malaria Consortium – Africa Regional Office, Kampala, Uganda; 4 London School of Hygiene and Tropical Medicine, London, United Kingdom; 5 Christoffel-Blindenmission (CBM), Juba, Southern Sudan; University of California San Francisco, United States of America

## Abstract

**Background:**

Trachoma is thought to be common over large parts of Southern Sudan. However, many areas of the country, particularly west of the Nile, have not yet been surveyed. The aim of this study was to confirm whether trachoma extends into Western Equatoria State from neighboring Central Equatoria, where trachoma is highly prevalent, and whether intervention with the SAFE strategy is required.

**Methods and Findings:**

Population-based cross-sectional surveys were conducted using a two-stage cluster random sampling method to select the study population. Subjects were examined for trachoma by experienced graders using the World Health Organization (WHO) simplified grading scheme. Two counties thought to be most likely to have trachoma were surveyed, Maridi and Mundri. In Maridi, prevalence of one of the signs of active trachoma (trachomatous inflammation-follicular (TF)) in children aged 1–9 years was 0.4% (95% confidence interval (CI), 0.0%–0.8%), while no children showing the other possible sign, trachomatous inflammation-intense (TI), were identified. No trachomatous trichiasis (TT) was found in those aged under 15, and prevalence was 0.1% (95% CI, 0.0%–0.4%) in those aged 15 years and above. In Mundri, active trachoma was also limited to signs of TF, with a prevalence of 4.1% (95% CI, 1.4%–6.9%) in children aged 1–9 years. Again, no TT was found in those aged under 15, and prevalence in those aged 15 years and above was 0.3% (95% CI, 0.0%–0.8%).

**Conclusion:**

Trachoma prevalence in the east of Western Equatoria State is below the WHO recommended intervention threshold for mass drug administration of antibiotic treatment in all villages. However, the prevalence of TF and TT in some villages, particularly in Mundri County, is sufficiently high to warrant targeted interventions at the community level. These results demonstrate that trachoma is not a major public health problem throughout Southern Sudan. Further studies will be required to determine trachoma prevalence in other areas, particularly west of the Nile, but there are presently no resources to survey each county. Studies should thus be targeted to areas where collection of new data would be most informative.

## Author Summary

Baseline data on trachoma prevalence is a prerequisite for intervention. Prior to the present study, all surveys in Southern Sudan reported trachoma prevalences that exceeded the threshold for large-scale intervention. This gave rise to the notion that the disease may be endemic throughout the country. The present study was conducted under the auspices of the National Program for Integrated Control of Neglected Tropical Diseases, to verify whether prevalences in two counties west of the Nile exceeded the WHO recommended intervention threshold for mass drug administration (MDA) of antibiotic treatment. The results show that trachoma prevalence at county level was below this threshold. However, prevalences in some communities within the county were above the threshold, meaning that they should be targeted with MDA of antibiotics, as well as with other interventions such as trichiasis surgery, health promotion and improved water and sanitation. This finding reminds us of the need for geographical targeting of resources, both for surveys and subsequent intervention. Current resources are insufficient to conduct population-based prevalence surveys for trachoma throughout Southern Sudan. Further surveys should thus be conducted in areas where collection of additional information will be most informative. We propose that a combination of risk-mapping and rapid assessments is used to identify such areas.

## Introduction

Trachoma is caused by ocular infection with the obligate intracellular bacterium *Chlamydia trachomatis*. Ocular Chlamydia is spread through contact with eye discharge from the infected person and through transmission by eye-seeking flies [1]. The disease is associated with poor personal and environmental hygiene, in particular limited access to water and sanitation, overcrowding and poor socioeconomic conditions.

Trachoma is the leading infectious cause of blindness, estimated to be responsible for 3.6% of blindness worldwide [2]. It is endemic in 56 countries, mainly in poor rural areas, including parts of Central and South America, many African countries and some countries in the Eastern Mediterranean [3]. However, there is a lack of information from some major populations, including large parts of Southern Sudan, which remains an important obstacle to estimating the disease burden and to the implementation of control efforts [4]. The World Health Organization (WHO) has identified a need for more trachoma data, and it is recognized that such data are necessary for implementation of the SAFE strategy: Surgery for trichiasis, Antibiotics to treat infection, and Facial cleanliness and Environmental improvement to reduce transmission [5].

Trachoma has long been known to be prevalent in parts of Sudan [6],[7], but comprehensive data on distribution and burden particularly in Southern Sudan continue to be limited. To generate baseline data, the Carter Center and the Ministry of Health, Government of Southern Sudan (MoH-GoSS), have jointly conducted prevalence surveys in thirteen sites covering a large geographical area mostly to the east of the river Nile [8],[9],[10],[11]. In all of these locations the average prevalence of active trachoma (trachomatous inflammation-follicular (TF) and/or trachomatous inflammation-intense (TI)) in children aged 1–9 years was found to be well above the 10% threshold recommended by WHO for large-scale SAFE intervention [5],[11],[12]. Ngondi and colleagues have used prevalence data from Upper Nile and Jonglei to estimate that in these two States alone 3.9 million people need antibiotic treatment and 206,000 people are in need of immediate trichiasis surgery [9]. These estimates are based on the assumption that trachoma prevalence is homogenous over large areas, which remains to be confirmed for Southern Sudan.

In common with other neglected tropical diseases (NTDs) endemic to Southern Sudan, there is a need to conduct additional surveys to better understand the epidemiology of trachoma and identify areas requiring interventions [13]. In Southern Sudan, a National Program for Integrated Control of NTDs has recently been established, presenting a new opportunity to contribute towards trachoma control by integrating annual distribution of antibiotic treatment with mass drug administration (MDA) of preventive chemotherapy (PCT) for other common NTDs, namely onchocerciasis, lymphatic filariasis (LF), soil-transmitted helminth (STH) infection and schistosomiasis [14],[15]. The program has started to operate in geographic areas where community-directed treatment with ivermectin (CDTI) for onchocerciasis control has been conducted for a number of years, because it intends to use the CDTI approach for co-implementation of other interventions where feasible [16],[17].

Western Equatoria State is one of the two States that has been selected for initial integrated NTD intervention, because it has a large onchocerciasis focus and a well-established CDTI network, and anecdotal and past survey data indicate that LF, STH infection and schistosomiasis are also prevalent [12]. To date there are, however, no information or data available for this State on the distribution or prevalence of trachoma. The present study was therefore conducted to generate baseline data on the prevalence of trachoma in parts of Western Equatoria State to provide evidence as to whether annual MDA of PCT should include antibiotic treatment, and to contribute data to revise mapping of the burden of trachoma in the region [4].

## Methods

### Study Site and Population

**Figure 1:**
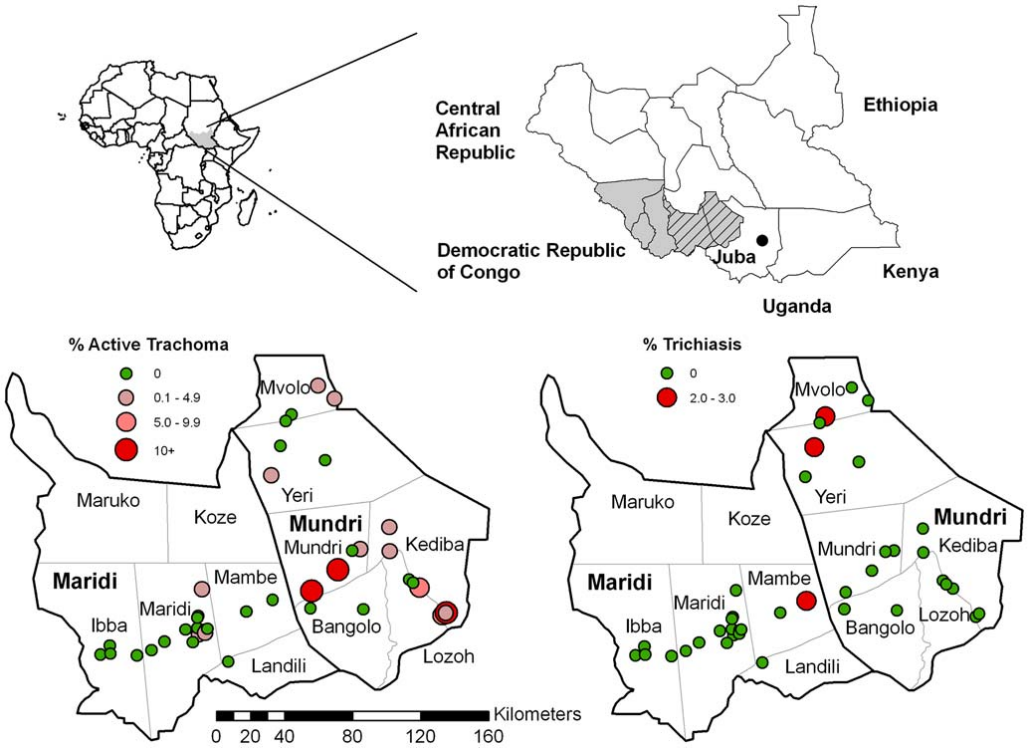
Maps showing surveyed counties in Western Equatoria State (hatched), and the prevalence of active trachoma (TF) and of trichiasis at each study site.

During November 2008, two population-based prevalence surveys were conducted in Western Equatoria State, which lies in the South-West of Southern Sudan (Figure 1). The majority of people are agriculturalist, growing maize, cassava, groundnut, and fruit. The state capital is Yambio, and the other major towns are Tambura, Nzara, Maridi and Mundri. The total population of Western Equatoria State was estimated to be 845,989 in 2008, using data collected during National Immunization Day. This is approximately 8% of the total population of Southern Sudan, which is estimated to be around 10 million. One trachoma survey was conducted in Mundri county and the other in Maridi county (Figure 1). At the time of the surveys, 186,668 people were estimated to live in Mundri and 186,830 in Maridi; both counties consisted of six payams.

The study followed the standard MoH-GoSS protocol for trachoma prevalence surveys [18]. The protocol recommends that population-based prevalence surveys are conducted at county (rather than payam) level, which is the second (rather than the third) administrative level; the State being the first administrative level in Southern Sudan. Estimation at county rather than payam level was thought to be consistent with WHO guidelines, which recommend trachoma prevalence be estimated at district level or an administrative area corresponding to an average population size of 100,000 [19],[20].

It was estimated that in each county a total sample size of 2000 people (of all ages and sexes) was required. This allows for an estimated prevalence of 5% trachomatous trichiasis (TT) in adults aged 15 years and above (chosen because TT was likely to be the least prevalent indicator measured) within a precision of 2%, given a 95% confidence limit and a design effect of 2, and based on the assumption that adults aged 15 years and above comprise 50% of the population.

In each county 20 villages were sampled with probability proportional to the estimated population size of the payam, although not all of the payams in Maridi were accessible. Households were randomly selected using the sketch map and segmentation method [21]. All residents of the household were enumerated, and all those present who gave informed consent were examined.

### Trachoma Grading

Ophthalmic Clinical Officers and General Clinical Officers from the local payams were trained by an experienced ophthalmologist (K. Lewis) to use the WHO simplified grading system [22]. This scheme categorizes trachoma infection according to five grades: TF, TI, trachomatous scarring (TS), TT and corneal opacity (CO). Two stages of assessment were used to select the best trainees. In the first stage, trainee examiners identified trachoma grades using the WHO set of trachoma slides [22]. Those examiners who achieved at least 80% agreement then proceeded to the second stage of field evaluation. During field evaluation, a reliability study comprising 50 persons of varying age and sex were selected by the ophthalmologist to represent all trachoma grades. Each trainee examiner evaluated all 50 participants independently and recorded their findings on a pre-printed form. Inter-observer agreement was then calculated for each trainee using the ophthalmologists' observation as the “gold standard.” Only trainees achieving at least 80% inter-observer agreement after the field evaluation were included as graders.

All inhabitants of selected households who provided verbal consent were examined using a torch and a 2× magnifying binocular loupe. Each eye was first examined for in-turned lashes (TT), and the cornea was then inspected for CO. The upper conjunctiva was subsequently examined for inflammation (TF and TI) and scarring (TS). Both eyes were examined. Signs had to be clearly visible in accordance with the simplified grading system in order to be considered present. Trachoma signs only had to be present in one eye for the person to be categorized as suffering from a particular grade of trachoma. Alcohol-soaked cotton swabs were used to clean the examiner's fingers between examinations. Individuals with signs of active trachoma or bacterial conjunctivitis were treated with 1% tetracycline eye ointment and provided with information on face washing and good hygiene practices. Patients with TT or other significant eye conditions were referred to the nearest facility where free surgery is available (i.e. Juba Teaching Hospital).

### Quality Control, Data Entry, and Analysis

The data was initially entered using Personal Digital Assistants (PDA, Palm Tungsten E2) in the field. A second data entry was conducted by the Trachoma Control Program, MoH-GoSS using Microsoft Office Excel. Consistency checks were performed in EpiInfo version 3.2.2 (Centers for Disease Control and Prevention [http://www.cdc.gov/EpiInfo]). Range and consistency checks were conducted for all variables. Data were analyzed in STATA 9.0 software (Stata Corporation, College Station, TX, U.S.A.). Individuals with missing data on sex and/or age were excluded from the analysis.

For each county, prevalences of trachoma signs were summarized by age, in relation to WHO recommendations for implementation of trachoma control activities [5]. Unadjusted exact binomial 95% confidence intervals (CIs) are presented, along with adjusted 95% CIs that account for potential clustering obtained using generalized estimating equation (GEE) modeling. Chi-squared tests, or Fishers test where appropriate, were used to examine evidence for differences in proportions.

### Ethical Considerations

The study protocol received ethical approval from the Directorate of Research, Planning and Health System Development, MoH-GoSS. Clearance to conduct the surveys was obtained from the State MoH, followed by County Health Departments and the local government. The study was explained to each member of the selected households. The household heads were asked to provide written consent for the entire household to participate in the study, and each inhabitant of the household who provided verbal consent was examined. Those individuals who did not provide verbal consent were not examined. Personal identifiers were removed from the dataset before analysis.

## Results

In the 20 study villages in each county, the number of households sampled and individuals examined were similar (Table 1). The average household size in Mundri was larger (p<0.001). There were three missing values for sex in Mundri. Age data were missing for approximately 10% of individuals in each county. There was no difference in the sex distribution by county (p = 0.672), whereas age distribution did differ by county (p<0.001), with more children aged 1–9 years and fewer people aged 15 or above in Maridi, compared to Mundri.

**Table 1:** Study population.

	Maridi	Mundri	p-Value*
Villages sampled	20	20	
Households sampled	393	400	
Median household size (range)	8 (1–26)	10 (2–30)	<0.001
Individuals enumerated	2702	3547	
Individuals enumerated with age and sex data†	2350	3190	
Male	1132	1555	
<1 year, n (%)	42 (3.7)	66 (4.2)	<0.001
1–9 years, n (%)	598 (52.8)	663 (42.6)	
10–14 years, n (%)	112 (9.9)	190 (12.2)	
15 & above, n (%)	380 (33.6)	636 (40.9)	
Female	1218	1635	
<1 year, n (%)	38 (3.1)	59 (3.6)	0.053
1–9 years, n (%)	526 (43.2)	625 (38.2)	
10–14 years, n (%)	120 (9.9)	162 (9.9)	
15 & above, n (%)	534 (43.8)	789 (48.3)	
Individuals examined‡			
Active trachoma signs (1–9 years)			
Male	532 (89.0)	523 (78.9)	
Female	464 (88.2)	503 (80.5)	
Trachomatous scarring (all ages)			
Male	895 (79.1)	1017 (65.4)	
Female	975 (80.0)	1225 (74.9)	
Trichiasis and corneal opacity (≥15 years)			
Male	268 (70.5)	348 (54.7)	
Female	422 (79.0)	579 (73.4)	

*p-value from chi-squared test.

†3 missing values for sex in Mundri, 352 (13.0%) missing values for age in Maridi, 353 (10.0%) missing values for age in Mundri. Age distribution based on sex denominators by county.

‡data are n (%) of enumerated individuals by sex and county.

Consent refusal was very low; 48 individuals (2.8%) refused overall, of which 16 (2.5%) were in Maridi and 32 (2.9%) in Mundri, although some enumerated individuals were subsequently unavailable for examination. Approximately 90% and 80% of children aged 1–9 from study villages in Maridi and Mundri were examined for signs of active trachoma, respectively. Examination data for TS were available for 80% of individuals in Maridi and 70% in Mundri. Of those aged 15 and above, TT and CO examinations were conducted in 75% of individuals in Maridi and 65% in Mundri.

### Active Trachoma

The overall prevalence of TF (adjusted 95% CI) in children aged 1–9 years in Maridi was 0.4% (0.0–0.8%) with cases occurring only in male children (Table 2). In Mundri, the prevalence of TF (adjusted 95% CI) in children aged 1–9 years was 4% (1.4–6.9%), with similar prevalence in male and female children. No cases of TI were found in either county, so prevalence estimates for active trachoma are the same as for TF.

**Table 2:** Prevalence of signs of trachoma.

Trachoma Signs	Maridi			Mundri		
	Number (%) of those examined	Unadjusted* 95% CI	Adjusted† Prevalence (95% CI)	Number (%) of those examined	Unadjusted* 95% CI	Adjusted† Prevalence (95% CI)
TF (1–9 years)						
Overall	4 (0.4)	0.1–1.0	0.4 (0.0–0.8)	41 (4.0)	2.9–5.4	4.1 (1.4–6.9)
Male	4 (0.8)	0.2–1.9	0.7 (0.0–1.6)	22 (4.2)	2.7–6.2	4.4 (0.8–8.0)
Female	0 (0)	0.0–0.8‡	-	19 (3.8)	2.3–5.8	3.8 (1.2–6.4)
TI (1–9 years)						
Overall	0 (0)	0.0–0.4‡	-	0 (0)	0.0–0.4‡	-
TS (<15 years)	0 (0)	0.0–0.3‡	-	1 (0.1)	0.0–0.4	0.0 (0.0–0.2)
TS (≥15 years)	1 (0.1)	0.0–0.8	0.1 (0.0–0.4)	8 (0.9)	0.4–1.7	0.9 (0.2–1.5)
TT (<15 years)	0 (0)	0.0–0.3‡	-	0 (0)	0.0–0.3‡	-
TT (≥15 years)	1 (0.1)	0.0–0.8	0.1 (0.0–0.4)	3 (0.3)	0.0–0.9	0.3 (0.0–0.8)
CO§ (<15 years)	0 (0)	0.0–0.3‡	-	0 (0)	0.0–0.3‡	-
CO§ (≥15 years)	1 (0.1)	0.0–0.8	0.1 (0.0–0.4)	3 (0.3)	0.0–0.9	0.3 (0.0–0.8)

TF = follicular trachoma, TI = inflammatory trachoma, TS = trachomatous scarring, TT = trichiasis, CO = corneal opacity.

*Exact binomial confidence intervals.

†Adjusted estimates obtained using generalised estimating equation (GEE) modelling.

‡97.5% one-sided confidence interval; GEE did not converge.

§Trachoma related corneal opacity: only participants with CO and TT were considered to have trachoma related CO.

### Trachomatous Trichiasis and Corneal Opacity

For TT, the overall prevalence (adjusted 95% CI) in those aged 15 years and above in Maridi was estimated to be 0.1% (0.0–0.4%) based on just one female individual with TT (Table 2). In Mundri, three male individuals were found to have TT leading to a prevalence estimate of 0.3% (0.0–0.8%). The small number of individuals with TT also had some degree of corneal opacity. No trichiasis was found in individuals under the age of 15 in either county.

### Trachomatous Scarring

Little evidence of scarring was observed in either county, the highest prevalence (adjusted 95% CI) was in individuals aged 15 and over in Mundri; 0.9% (0.2–1.5%).

### Intervention Implications

No villages in Maridi had a prevalence of TF above 5%, but one village did have a prevalence of TT of more than 1% (Table 3, Figure 1). In Mundri, two villages had a prevalence of TF between 5% and 9% and a further three villages had a prevalence of more than 10% (Table 3, Figure 1), suggesting a requirement for F and E components of the SAFE strategy in these five villages along with annual MDA of antibiotics in the three villages with higher prevalence. Two additional villages in Mundri had a prevalence of TT of more than 1% (Figure 1).

**Table 3:** Community prevalence by county.

Villages sampled		Maridi		Mundri	
		20		20	
		n (%)	Prevalence if above threshold*	n (%)	Village prevalence if above threshold*
TF in children 1–9 years	0%	17 (85.0%)	-	8 (40.0%)	-
	<5%	3 (15.0%)	-	7 (35.0%)	-
	5–9%	0 (0)	-	2 (10.0%)	5.4%, 7.8%
	10–19%	0 (0)	-	2 (10.0%)	13.0%, 16.3%
	20–25%	0 (0)	-	1 (5.0%)	22.9%
TT in those 15 years & above	0%	19 (95.0%)	-	19 (90.0%)	-
	<1%	0 (0)	-	0 (0)	-
	≥1%	1 (5.0%)	2.1%	2 (10.0%)	3.0%, 3.5%

TF = follicular trachoma, TT = trichiasis.

*Threshold for implementation of trachoma control activities according to WHO guidelines; i.e. implementation of trachoma control activities is prioritized in communities where the prevalence of active trachoma in children aged 1–9 years is 10% or higher or where the prevalence of trichiasis in people aged 15 years and over is 1% or higher [5].

## Discussion

This study was conducted within the context of scaling up of the National Program for Integrated Control of NTDs, to decide whether antibiotic treatment for trachoma should be included in annual MDA of PCT for other NTDs in Western Equatoria State. Based on consultation with State and County Health Authorities, two counties were selected to represent those areas of the State thought to be worst affected by trachoma. Despite purposefully selecting these areas, we found that the overall burden of active trachoma in children aged 1–9 years was below the 10% threshold recommended by WHO for large-scale MDA of antibiotic treatment. Similarly, the overall prevalence of TT in people aged 15 years or over was below the recommended 1% threshold for large-scale SAFE intervention. However, some study villages in Mundri County did have TF prevalences that exceeded 5%, which warrants community-wide intervention by means of annual MDA of antibiotic treatment, health promotion and improvements of water and sanitation [5]. The three study communities with cases of trichiasis will need to be provided with access to surgery. It is likely that other communities, particularly in Mundri County, are also affected by active trachoma and/or TT and need to be provided with some or all components of the SAFE strategy. Community-by-community assessments will be needed to identify these [5].

The relatively low trachoma prevalences observed in Maridi and Mundri are in stark contrast to those reported by investigators who surveyed other parts of Southern Sudan, including two sites (Tali and Katigiri) adjacent to Mundri that were found to have 72.6% and 50.0% prevalence of active trachoma in children aged 1–9, respectively [9]. In fact, in all other sites previously surveyed throughout Southern Sudan, prevalence of active trachoma exceeded the 10% intervention threshold by up to eight times [8]–[11]. Present findings thus provide an important contribution to our understanding of trachoma epidemiology in Southern Sudan, showing that the disease is not highly endemic throughout the country and that prevalence can vary considerably between adjacent counties. Based on this observation it would be unwise to conclude from our data that the whole of Western Equatoria State has a low prevalence of trachoma, but given that we purposefully selected areas that were thought to be worst affected, we may assume this is the case. To verify this assumption and to decide whether further population-based prevalence surveys are required in this State and elsewhere, we propose that more evidence on the distribution and burden of trachoma is collected through a combination of risk-mapping, using a global information system (GIS), and rapid assessments [23]. For the purpose of risk-mapping, existing trachoma prevalence data for Southern Sudan, as well as environmental and other potential risk factors [23], should be incorporated into a model to predict areas at risk of trachoma transmission. Although GIS has so far not been used to predict the spatial distribution of trachoma, the approach has informed targeting of a number of other NTD interventions, such as schistosomiasis [24]–[26]. By taking account of the specific epidemiological characteristics of trachoma, it may be possible to develop a GIS-based model that could complement the existing methods for identifying trachoma endemic areas [27].

The challenging operating conditions in Southern Sudan meant that survey implementation deviated slightly from the approach recommended in the national trachoma survey protocol [18]. Due to a lack of security not all of the payams in Maridi were accessible. In both counties we had to exclude some of the randomly selected village, because they were inaccessible due to flooding or impassable roads, or we found that they were more than a 45-minute walk from the nearest vehicle access point. Furthermore, some villages that were recorded on the maps developed by the United Nations Joint Logistics Coordination and International Mapping Unit no longer existed. As we had used these maps to select villages, it meant that not all of the sites from our initial random selection could be surveyed. If a village was known to be inaccessible or non-existent in advance, an alternative site was randomly selected. If the village was found to be inaccessible or non-existent on arrival, an alternative site close to that geographic location was selected.

This slight deviation from a true random selection of villages may have introduced bias, thus affecting our estimation of overall trachoma prevalence in a county. For example, overall prevalence may have been underestimated if the villages inaccessible at the time of the survey had poorer access to healthcare, so that residents were less likely to have been treated for trachoma. However, our field observations indicated that healthcare provision was uniformly poor across the survey area, with no major variations in access or quality between villages. Healthcare delivery largely consisted of community health workers with little equipment or training. Because few of the randomly selected villages were excluded, we consider it unlikely that any significant bias was introduced to the overall estimate of trachoma prevalence for either county.

Of the individuals living in households recruited into the study, the overall percentage examined was 68.9% in Mundri and 76.2% in Maridi. This rate was somewhat less than that reported by other investigators, who examined 85%–90% of registered household occupants during surveys in other parts of Southern Sudan [10],[11],[28]. The main reason for non-response was that individuals were not present at the time of the survey; unfortunately it was not possible to follow these up due to the difficult operating conditions. The majority of those absent were men aged 15 years and above, meaning that significantly more females than males in older age groups were examined in both counties. Such absenteeism may have biased our overall findings, possibly resulting in an overestimate of trachoma prevalence if, for example, males with good eyesight had been more able to leave home. We were unable to investigate this further, because trachoma was not sufficiently endemic to make a valid comparison of prevalence between males and females. Lastly, we had to exclude some incomplete eye examinations from the analysis, as some individuals withdrew their consent after examination of the first eye. This was more common in children, and might have led to an underestimation of TI and TF infection, particularly if infected individuals experienced more discomfort during examination and were thus more likely to withdraw their consent.

Despite these operational limitations and their potential implications for the precision of our estimates, the low trachoma prevalence found clearly shows that annual MDA with antibiotics does not need to be targeted at all villages in the two counties, but only at a selection within these. This means that integration of trachoma treatment as part of annual MDA for the other diseases targeted by the National Program for Integrated Control of NTDs would not be practical.

There are a number of possible explanations for the difference in the results of the present study when compared to those of other investigators. Firstly, previous surveys on trachoma in Southern Sudan were conducted in areas where health workers had already reported trachoma to be a cause of blindness. In contrast, the selection of the present study sites was not based on such reports, but on the assessment of State and County health staff as to which areas are worst affected by trachoma, in a State that generally experiences little demand for trachoma treatment services. This difference in the selection of the survey area will have affected our results. Whereas investigators of previous studies had good evidence that trachoma was endemic before they undertook the surveys, there was no indication that it constituted a major public health problem in Mundri or Maridi.

The second difference between the present study and those previously conducted in Southern Sudan is that we used different survey staff. The graders used in the present study may have under-graded symptoms, when compared to grading done in previous studies, which in turn may have led to systematic bias in clinical grading. This could explain why there were seemingly no cases of active trachoma in villages with TT and vice versa (Figure 1). Though we can not exclude this possibility, it may also be possible that these findings are the result of extensive population movement in Southern Sudan, with the few diagnosed TT cases maybe having moved relatively recently from trachoma endemic areas to these three villages. It is unlikely that the absence of active cases in villages with TT is a result of recent socioeconomic development and associated improvements in access to safe water and proper sanitation, as such development is proceeding very slowly throughout the country.

To minimize the possibility of under- or over-grading of trachoma symptoms, we ensured that the clinical scoring conducted by our graders was consistent and agreed with a standardized set of photos. We also assessed the feasibility of verifying the graders findings by taking photographs and/or samples for analysis by nucleic acid amplification tests as suggested elsewhere [29], but established that both methods were beyond what was feasible with existing resources and in an environment with no infrastructure. For future trachoma studies, the possibility of implementing either or both of these two complementary approaches should be reassessed and budgeted for.

The third potential variation between the current and previous surveys is a difference in the livelihoods between the populations sampled, as well as their hygiene behavior. A detailed analysis of trachoma risk factors in the previously sampled populations [30] showed that cattle ownership was common (69.2% overall), and increased the risk of trachoma infection, along with other factors such as unclean face, face washing less than twice a day and high household fly density. The geographical area visited during the present surveys, however, is known to be largely inhabited by agriculturalists. This was confirmed in our household surveys, which showed that 86.7% and 75.3% of households in Maridi and Mundri, respectively, were mainly involved in agriculture (data not shown). The absence of large amounts of livestock and their dung might have resulted in lower fly densities and hence less transmission. In addition, Ngondi and colleagues [30] reported anecdotal evidence that people keeping cattle also dispose of human feces in the cattle pens, hence providing an even more favorable breeding environment for flies. As we did not investigate behavior in sufficient detail, this and other potential differences in daily hygiene and sanitation behavior between agriculturalist and pastoralist tribes in Southern Sudan remains hypothetical. Further sociological studies would help to better understand such differences and their potential implications on trachoma transmission [30].

Above explanations are likely to account for only part of the differences between study sites in Southern Sudan. The importance of geographical, climatic and socio-economic determinants, for example, warrants further investigation. Their possible association in Southern Sudan has not been sufficiently researched, although an earlier study in the Sudan provides evidence for an inverse correlation of TF/TI prevalence with humidity and rainfall [7].

### Conclusion

The low prevalence of trachoma observed in the present study and the lack of reported cases from other counties in Western Equatoria State suggest that trachoma is also unlikely to be highly endemic in those counties not surveyed to date. Although this needs to be verified, we do not consider this a current priority. Instead, the limited resources available for surveys should be used to generate baseline data for counties where trachoma is known to be a problem, but where there is no data to monitor interventions. Among these, counties where intervention is feasible (in terms of access and funding) should be prioritized.

In addition, it will be important to complete the picture of trachoma epidemiology in Southern Sudan, to get a better understanding of the scale of the burden and the resources required to eliminating blinding trachoma from Southern Sudan by 2020. In the interest of conserving scarce resources, we propose that trachoma distribution is assessed in stages. In the first stage, the large amount of existing prevalence data should be used to develop a trachoma risk map for the whole of Southern Sudan, which will not only indicate where trachoma is certain to be highly endemic but also where prediction of endemicity is rather uncertain. Based on this information, rapid assessments should then be conducted in those areas with no data and high uncertainty of the level of endemicity. Based on the outcome of such assessments it can be decided whether one should move to the third stage - detailed prevalence surveys - to provide baseline data. In an iterative process, the data generated through rapid assessments and additional prevalence surveys should be used to verify the predictions of the risk map model, and fine tune and improve these, including estimates of the national trachoma burden.

## Supporting Information

Checklist S1 STROBE checklist.
(0.08 MB DOC)
